# Gray Matter Atrophy in Amnestic Mild Cognitive Impairment: A Voxel-Based Meta-Analysis

**DOI:** 10.3389/fnagi.2021.627919

**Published:** 2021-03-31

**Authors:** Jinhuan Zhang, Yongfeng Liu, Kai Lan, Xingxian Huang, Yuhai He, Fuxia Yang, Jiaying Li, Qingmao Hu, Jinping Xu, Haibo Yu

**Affiliations:** ^1^The Fourth Clinical Medical College of Guangzhou University of Chinese Medicine, Shenzhen, China; ^2^Institute of Biomedical and Health Engineering, Shenzhen Institutes of Advanced Technology, Chinese Academy of Sciences, Shenzhen, China; ^3^Department of Acupuncture and Moxibustion, Shenzhen Traditional Chinese Medicine Hospital, Shenzhen, China

**Keywords:** amnestic mild cognitive impairment, voxel-based morphometry, gray matter volume, meta-analysis, seed-based *d* mapping

## Abstract

**Background:** Voxel-based morphometry (VBM) has been widely used to investigate structural alterations in amnesia mild cognitive impairment (aMCI). However, inconsistent results have hindered our understanding of the exact neuropathology related to aMCI.

**Objectives:** Our aim was to systematically review the literature reporting VBM on aMCI to elucidate consistent gray matter alterations, their functional characterization, and corresponding co-activation patterns.

**Methods:** The PubMed, Web of Science, and EMBASE databases were searched for VBM studies on aMCI published from inception up to June 2020. Peak coordinates were extracted from clusters that showed significant gray matter differences between aMCI patients and healthy controls (HC). Meta-analysis was performed using seed-based *d* mapping with the permutation of subject images (SDM-PSI), a newly improved meta-analytic method. Functional characterization and task-based co-activation patterns using the BrainMap database were performed on significant clusters to explore their functional roles. Finally, VBM was performed based on the Alzheimer's Disease Neuroimaging Initiative (ADNI) dataset to further support the findings.

**Results:** A total of 31 studies with 681 aMCI patients and 837 HC were included in this systematic review. The aMCI group showed significant gray matter atrophy in the left amygdala and right hippocampus, which was consistent with results from the ADNI dataset. Functional characterization revealed that these regions were mainly associated with emotion, cognition, and perception. Further, meta-regression analysis demonstrated that gray matter atrophy in the left inferior frontal gyrus and the left angular gyrus was significantly associated with cognitive impairment in the aMCI group.

**Conclusions:** The findings of gray matter atrophy in the left amygdala and right hippocampus are highly consistent and robust, and not only offer a better understanding of the underlying neuropathology but also provide accurate potential biomarkers for aMCI.

## Introduction

Mild cognitive impairment (MCI) is a transitional stage between normal cognitive function and dementia, mainly characterized by mild impaired cognitive function but without significant impairment of function (Bennett et al., 2002; Petersen, 2004). Amnestic MCI (aMCI), is a subtype of MCI characterized by objective memory impairment without dementia, preserved general cognitive function, and highly intact functional activities (Petersen, 2004). aMCI conveys a high risk for developing Alzheimer's disease (AD), with an annual rate of ~25% of patients (Petersen et al., 2001b; Zhao et al., 2014). Although numerous studies have reported gray matter atrophy in many brain regions and have suggested it to be associated with the pathophysiology of aMCI, the results are inconsistent (Threlkeld et al., 2011; Baglio et al., 2012; Migo et al., 2015), and need to be verified.

In recent years, numerous systematic reviews have been performed to analyze the difference in gray matter between MCI and HC (Schroeter et al., 2009; Shi et al., 2009; Yang et al., 2012; Tabatabaei-Jafari et al., 2015; Minkova et al., 2017; Gu and Zhang, 2019). However, the studies showed that MCI, including both aMCI and non-aMCI, is a heterogeneous clinical identity displaying the loss of different neurodegenerative entities (Costafreda et al., 2009; Serra et al., 2013), thus, should be studied at the subtype level. The most recent meta-analysis (Nickl-Jockschat et al., 2012) on the gray matter differences between aMCI and HC was conducted in 2012, and revealed significant atrophy in the bilateral amygdala, hippocampus, left superior temporal gyrus, and the left thalamus. An increasing number of voxel-based morphometry (VBM) studies have investigated gray matter differences between aMCI and HC. However, due to the small and heterogeneous samples of participants as well as substantial methodological differences between studies, results from VBM studies remain inconsistent and controversial (Costafreda et al., 2009). For example, some studies report that regional gray matter atrophy is mainly restricted to the bilateral hippocampus (Pa et al., 2009; Gili et al., 2011), whereas other studies only report gray matter volume (GMV) loss in the unilateral hippocampus (left or right) (Bonekamp et al., 2010; Xie et al., 2015). Moreover, a statistical method of the meta-analysis has been optimized (Albajes-Eizagirre et al., 2019a,b). A new-generation algorithm for coordinate-based meta-analysis (CBMA), seed-based *d* mapping with the permutation of subject images (SDM-PSI), has been successfully used in previous VBM meta-analysis studies (Albajes-Eizagirre et al., 2019b; Wang et al., 2020). This method has led to significant improvements, such as using threshold-free cluster enhancement (TFCE) statistics, small bias estimates of the overall size estimates, and multiple imputations of the study image, to avoid bias associated with single imputation (Albajes-Eizagirre et al., 2019a).

Therefore, in this systematic review of VBM studies, SDM-PSI was used to determine the most prominent and replicable areas that can distinguish aMCI from healthy controls. Further, meta-analytic connectivity modeling (MACM) analysis was performed to understand the role of significance clusters at the functional network level. Behavioral domains (BD) and paradigm classes (PC) were used to determine functional characterization of significance clusters. A dataset (144 aMCI and 87 HC) from the Alzheimer's Disease Neuroimaging Initiative (ADNI) database was used to compare gray matter atrophy of aMCI and HC to further validate the results of our meta-analysis.

## Materials and Methods

The meta-analysis was conducted in accordance with the Preferred Reporting Items for Systematic Reviews and Meta-analyses (PRISMA) statement (Moher et al., 2009; Muller et al., 2018) (Supplementary Table 1). The present meta-analysis was undertaken following the recent guidelines and recommendations for CBMA (Winblad et al., 2004; Tahmasian et al., 2019). The protocol of this meta-analysis was registered at PROSPERO (http://www.crd.york.ac.uk/PROSPERO) (registration number: CRD42020204050).

### Literature Search and Study Selection

A systematic search strategy was conducted in PubMed, EMBASE, and Web of Science (https://www.webofknowledge.com/) from inception to June 2020. The search keywords used were (“cognitive impairment” OR “mild cognitive impairment” OR “cognitive decline” [Title/Abstract]) OR “neurocognitive disorder” OR “MCI”) AND (“voxel-based morphometry” OR “VBM” OR “morphometry”) OR “volumetry” OR “gray matter” OR “structural MRI”). Besides, the references of the included studies were manually screened to avoid omission of relevant studies, and all the identified studies were imported into EndNote. After a review of the title and abstracts, studies that did not meet the inclusion criteria were excluded. A final exclusion of studies was performed after a full-text review.

**Figure 1:**
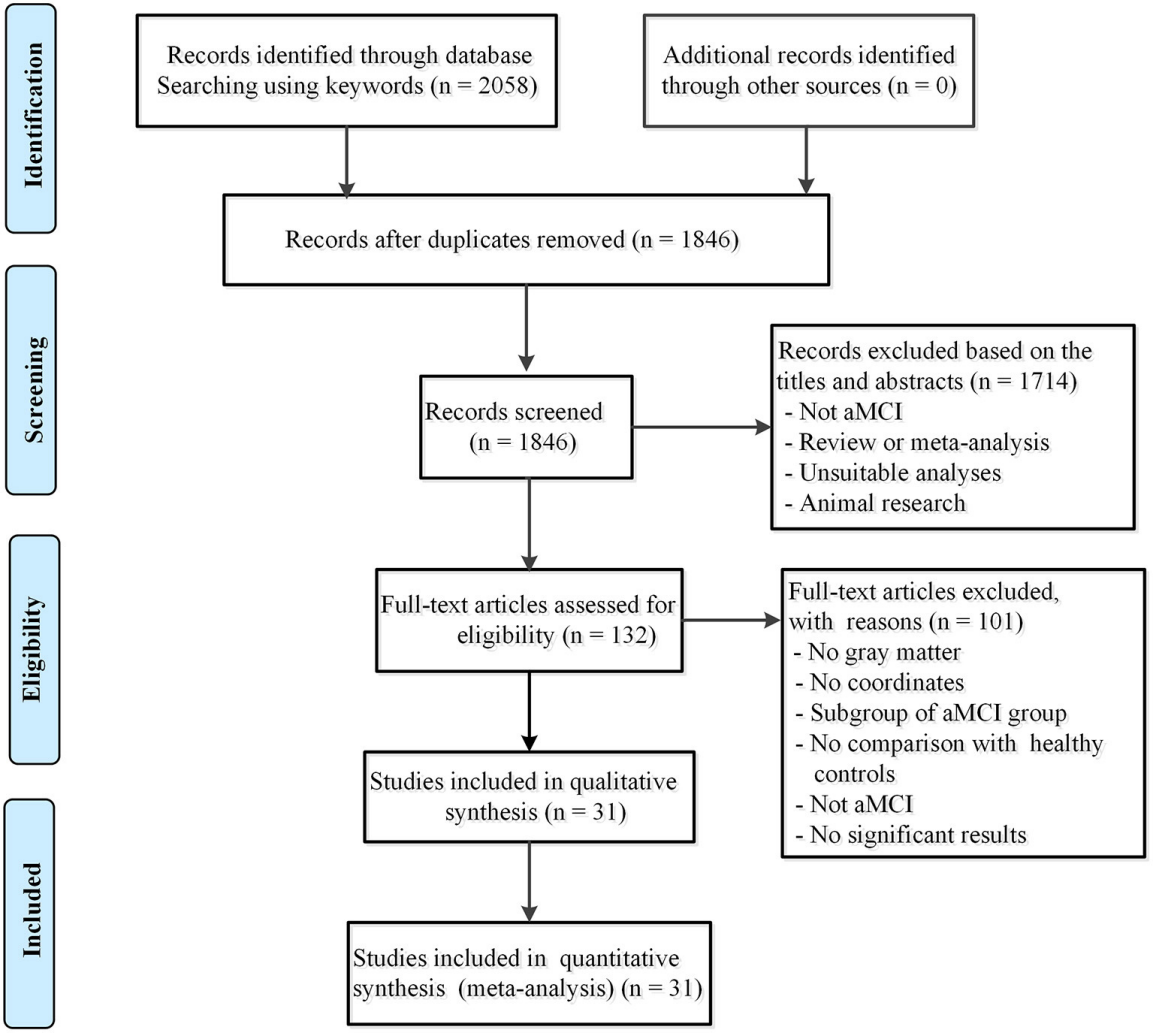
Flow diagram of inclusion and exclusion process of selected articles of VBM studies in patients with aMCI. aMCI, amnestic mild cognitive impairment.

Studies were included if they met the following criteria: (1) the patients met the clear diagnostic criteria for aMCI (McKhann et al., 1984; Petersen et al., 2001a,b; Petersen, 2004; Du et al., 2014); (2) the study utilized the VBM method to estimate GMV or differences in gray matter density at the whole-brain level between aMCI and HC; (3) the study used stereotactic coordinates (i.e., Talairach space or Montreal Neurological Institute (MNI) space); and (4) the study was an original article, peer-reviewed, and published in English. The appropriate results of the meta-analysis were based on the overall effect in the subgroups. Studies reporting aMCI patients with other neurological, psychiatric, or systemic diseases or postoperative complications were also excluded (i.e.; stroke, Parkinson's disease, or diabetes). For longitudinal design studies, only baseline preprocessing data were included. Authors of published studies were contacted for additional information by email. Following this approach, 31 studies were selected (Figure 1).

### Data Extraction and Quality Assessment

The extracted data included study characteristics (author and year of publication), subject characteristics [sample size, age, gender, education, and mini-mental state examination (MMSE)], peak coordinates, and the significance level. Moreover, coordinates in different stereotactic spaces were converted to MNI space, while Z- or *P*-values for significant clusters were converted to *T*-values using SDM utilities (https://www.sdmproject.com/utilities/?show=Statistics).

A 12-point checklist (Supplementary Table 2) containing the inclusion criteria, demographic characteristics, sample size, technical details of the imaging procedure, analysis method, and the quality of the reported results was assessed to determine the quality of each selected article (Radua et al., 2015). All the steps were independently performed by two authors, and all inconsistencies were resolved by a third author.

### Coordinate-Based Meta-Analysis

CBMA was carried out using SDM-PSI version 6.21 (https://www.sdmproject.com/), with the following procedure: (1) collection of information regarding the peak coordinates of significant GM differences between aMCI and HC; (2) calculation of the maps of the lower and upper bounds of possible effect sizes within a GM mask, full anisotropy = 1, isotropic full width half maximum (FWHM) = 20 mm, and voxel = 2 mm; (3) the mean analysis: estimation of the map of most likely effect size and its standard error based on the MetaNSUE algorithms (Radua et al., 2012; Albajes-Eizagirre et al., 2019b); (4) conducting multiple imputations of the maps of the effect size of individual studies; (5) meta-analysis of these maps using a standard random-effects model, and Rubin rules to pool the different meta-analyses resulting from the multiple imputations (Albajes-Eizagirre et al., 2019b); (6) family-wise error (FWE) correction for multiple comparisons; and (7) using threshold-free cluster enhancement (TFCE) in the statistical thresholding (*p* < 0.05, voxel extent ≥ 10). The details of these procedures have been extensively described in prior publications (Albajes-Eizagirre et al., 2019a,b) and the SDM-PSI reference manual (https://www.sdmproject.com/manual/).

### Reliability Analysis, Subgroup Meta-Analysis, and Meta-Regressions

To test the replicability of the results, a systematic whole-brain voxel-based jackknife sensitivity analysis was performed using the leave-one-out method (Radua and Mataix-Cols, 2009; Albajes-Eizagirre et al., 2019a).

Subgroup meta-analyses were performed to investigate the potential confounding effects. Imaging methodology variables including datasets using a 3.0 T MRI scanner, statistical parametric mapping (SPM) software versions 8 or 12, a smoothing kernel of 8 mm, and the corrected thresholds for multiple comparisons, respectively, were performed. Statistical significance was determined using the TFCE-based FWE corrected threshold (*p* < 0.05, voxel extent ≥ 10) (Radua et al., 2014; Albajes-Eizagirre et al., 2019b).

Finally, meta-regression analyses were conducted to examine the potential effects of demographic characteristics and clinical confounders (age, gender, education, and MMSE) on GMV by linear regression. Confounders were weighted based on the square root of the sample size and restricted to only predict possible SDM values (Radua et al., 2014). Statistical significance was determined using a stringent threshold of *p* < 0.05 and cluster extent ≥ 10 voxels in the meta-regression analyses (Higgins et al., 2003; Radua and Mataix-Cols, 2009).

### Analyses of Heterogeneity and Publication Bias

The values from peak coordinates reported in the CBMA were extracted for information to guide heterogeneity statistics and publication bias analyses. Heterogeneity between studies was assessed using the *I*^2^ statistic using a random-effects model, where *I*^2^ < 50% indicates low heterogeneity (Egger et al., 1997). Publication bias was examined using funnel plots and Egger tests (Eickhoff et al., 2011). An asymmetric plot and *p* < 0.05 were considered statistically significant.

### Analysis of Co-activation Patterns and Functional Characterization

To determine the role of these clusters at the functional network level, we performed MACM to obtain the task-dependent co-activated patterns of each brain region in the BrainMap database (http://brainmap.org/) by performing an activation likelihood estimation (ALE) (Eickhoff et al., 2009; Tench et al., 2014). The ALE scores were compared to a null-distribution of random spatial association between experiments with a fixed within-experiment distribution of foci (Eickhoff et al., 2012) yielding a *p*-value based on the proportion of equal or higher random values (Eickhoff et al., 2011). These non-parametric *p*-values were converted to z-scores and corrected at a cluster-level FWE-corrected threshold of *p* < 0.05 (a voxel-level *p* < 0.001).

Functional characterization determines the functional role of the brain region in terms of behavioral domains (BD) and paradigm classes (PC) using forward inference in the BrainMap database (Laird et al., 2009; Turner and Laird, 2012) (http://www.brainmap.org). Behavioral domains include the main categories of cognition, action, perception, emotion, and interception, as well as their related sub-categories. Paradigm classes classify the specific task employed (Muller et al., 2013) (http://brainmap.org/scribe/) for complete BrainMap taxonomy. Significance was assessed using a binomial test (*p* < 0.05, corrected for multiple comparisons using FDR) (Weiner et al., 2013).

### Alzheimer's Disease Neuroimaging Initiative Database

The gray matter differences between aMCI and HC were further studied using the ADNI database, to support the meta-analysis results. Inclusive and exclusive criteria are described in detail at http://www.adni-info.org.

Data were downloaded from the ADNI database up to July 2020 (Chetelat et al., 2002) (http://adni.loni.ucla.edu/). The search included insertion of MCI and HC in the research group and selecting MRI modalities from the ADNI.

The T1 images were preprocessed using DPABI (http://rfmri.org/dpabi). First, each image was segmented into gray matter, white matter, and cerebrospinal fluid, and the images were transformed to MNI space. The gray matter images were modulated to preserve regional volume information. Finally, the modulated images were smoothed with a 6 mm FWHM. Two-sample *t*-tests were performed to identify GMV differences between aMCI and HC. Age and sex were entered into the models as covariates. Results were corrected for Gaussian Random Field (GRF) with a voxel level of *p* < 0.001 and a cluster level of *p* < 0.05.

## Results

### Included Studies and Sample Characteristics

Thirty-one studies (Bell-McGinty et al., 2005; Hirata et al., 2005; Saykin et al., 2006; Shiino et al., 2006; Trivedi et al., 2006; Hämäläinen et al., 2007; Bai et al., 2008; Barbeau et al., 2008; Barnes et al., 2009; Guedj et al., 2009; Pa et al., 2009; Rami et al., 2009; Bonekamp et al., 2010; Agosta et al., 2011; Derflinger et al., 2011; Threlkeld et al., 2011; Venneri et al., 2011; Baglio et al., 2012; Han et al., 2012; Wang et al., 2012; Xie et al., 2012, 2015; Bastin et al., 2013; Hoppstädter et al., 2013; Serra et al., 2013; Zhao et al., 2014, 2015; Hong et al., 2015; Migo et al., 2015; Sheelakumari et al., 2018; Chen et al., 2020) were included comprising 681 aMCI patients (211 male and 207 female) and 837 HC (257 male and 301 female). An unbalanced age distribution was observed between aMCI and HC (standardized mean difference [SMD] = −0.20, 95% confidence interval [CI] = [−0.31, −0.10], z = 3.74, *p* < 0.01), and one dataset (Pa et al., 2009) did not report the mean age and standard deviation of HC. Significant differences were observed between aMCI and HC regarding gender (χ^2^ = 4.7, *p* = 0.03), however, the sex ratios of the two datasets (Saykin et al., 2006; Chen et al., 2020) were not provided. In terms of educational level, the aMCI group had fewer years of education compared with the HC group (SMD = −1.32, 95%CI = [−1.45, −1.18], z = 19.23, *p* < 0.01), the education levels in six datasets (Bell-McGinty et al., 2005; Saykin et al., 2006; Trivedi et al., 2006; Guedj et al., 2009; Pa et al., 2009; Agosta et al., 2011) were not provided. Besides, the aMCI groups had significantly lower MMSE scores than the HC group (SMD = −0.19, 95% CI = [-0.31, −0.07], z = 3.19, *p* < 0.01). The MMSE scores in seven datasets were not provided.

The quality of each included study (Supplementary Table 2) was acceptable, with a quality score not <10 (a maximum score =11.5). The demographic, clinical, and quality score of each eligible study are summarized in Table 1. Technical characteristics are shown in Supplementary Table 3.

**Table 1:** Demographic and clinical characteristics of the included VBM studies.

**Study**	**Gender (F/M)**		**Age (SD)**		**Education (SD)**		**MMSE (SD)**		**Quality scores (out of 12)**
	**HC**	**MCI**	**HC**	**MCI**	**HC**	**MCI**	**HC**	**aMCI**	
Chetelat et al. (2002), Chetelat et al. (2002), Bell-McGinty et al. (2005)	22 (12/10)	22 (12/10)	66.6 (7.2)	71.0 (8.0)	NA	NA	NA	NA	11
Bell-McGinty et al. (2005), Hirata et al. (2005)	47 (20/27)	9 (5/4)	66.9 (7.3)	71.9 (7.6)	15.7 (2.7)	13.7 (2.1)	29.4 (0.4)	23.1 (3.8)	10.5
Hirata et al. (2005), Saykin et al. (2006)	30	41	70.6 (8.4)	71.1 (7.7)	NA	NA	28.7 (1.5.)	26.0 (1.5)	10.5
Saykin et al. (2006), Shiino et al. (2006)	40 (28/12)	40 (17/23)	63.4 (8.9)	70.9 (9.0)	16.6 (2.7)	16.3 (3.3)	29.1 (1.0)	27.2 (2.2)	11.5
Shiino et al. (2006), Trivedi et al. (2006)	88 (48/40)	20 (10/10)	68.7 (8.7)	67.7 (9.0)	NA	NA	29.09 (1.47)	26.80 (1.88)	11
Trivedi et al. (2006), Hämäläinen et al. (2007)	15 (6/9)	15 (6/9)	73.6 (7.1)	73.3 (6.7)	16.7 (2.5)	16.3 (2.8)	29.7 (0.5)	27.8 (1.8)	11.5
Hämäläinen et al. (2007), Bai et al. (2008)	21 (17/4)	14 (10/4)	71.2 (4.9)	72.4 (7.3)	7.9 (2.9)	8.1 (2.6)	27.7 (2.0)	5.6 (3.1)	11
Bai et al. (2008), Barbeau et al. (2008)	20 (11/9)	20 (10/10)	69.4 (3.8)	71.3 (3.8)	13.8 (4)	14.0 (3.1)	28.3 (1.4)	27.2 (1.6)	11
Barbeau et al. (2008), Guedj et al. (2009)	28 (13/15)	28 (16/12)	63.3 (7.2)	69.3 (8.6)	NA	NA	28.9 (1.0)	27.4 (1.4)	11
Guedj et al. (2009), Pa et al. (2009)	28	19 (10/9)	NA	69.9 (9.5)	NA	NA	28.8 (1.0)	27.1 (1.1)	10
Pa et al. (2009), Rami et al. (2009)	36 (23/13)	26 (13/13)	64.8 (8.2)	68.0 (6.6)	17.0 (2.0)	17.5 (1.7)	NA	NA	11
Rami et al. (2009), Bonekamp et al. (2010)	27 (17/10)	14 (10/4)	74.3 (5.3)	72.9 (4.8)	9.4 (5.2)	7.4 (4.2)	27.4 (1.0)	26.0 (2.0)	11
Bonekamp et al. (2010), Agosta et al. (2011)	20 (10/10)	10 (5/5)	75.5 (4.6)	73.5 (5.5)	NA	NA	28.9 (1.2)	26.3 (2.9)	11.5
Agosta et al. (2011), Derflinger et al. (2011)	15 (9/6)	15 (7/8)	69.8 (6.0)	70.4 (7.2)	12.3 (3.6)	9.0 (4.6)	28.8 (1.5)	25.8 (0.9)	11.5
Derflinger et al. (2011), Threlkeld et al. (2011)	30 (20/10)	24 (13/11)	67.0 (8.7)	69.0 (9.0)	10.6 (1.7)	10.4 (2.0)	NA	26.8 (1.7)	11.5
Threlkeld et al. (2011)	24 (11/13)	18 (8/10)	77.9 (7.1)	77.1 (5.8)	16.2 (2.4)	15.8 (2.6)	28.4 (1.1)	27.1 (1.3)	11
Venneri et al. (2011), Venneri et al. (2011)	25 (15/10)	25 (12/13)	70.3 (6.5)	70.5 (6.4)	9.32 (4.46)	8.96 (4.41)	28.68 (1.52)	28.24 (1.23)	10.5
Baglio et al. (2012), Baglio et al. (2012)	15 (9/6)	16 (7/9)	71.0 (5.8)	66.9 (6.4)	10.8 (3.5)	9.9 (4.8)	29.0 (1.3)	27.0 (1.8)	10.5
Han et al. (2012), Han et al. (2012), Wang et al. (2012)	18 (11/7)	17 (10/7)	66.5 (6.2)	69.7 (7.6)	8.4 (5.6)	8.8 (4.0)	29.2 (0.7)	25.2 (3.5)	11
Wang et al. (2012), Xie et al. (2012)	30 (11/19)	40 (16/24)	76.1 (7.2)	68.07 (7.46)	13.5 (2.6)	11.4 (4.3)	NA	NA	11.5
Xie et al. (2012), Bastin et al. (2013)	25 (12/13)	17 (11/6)	74.3 (8.3)	75.1 (6.6)	15.3 (2.9)	13.5 (2.1)	28.9 (1.2)	27.3 (1.8)	11
Bastin et al. (2013), Hoppstädter et al. (2013)	24 (18/6)	35 (12/23)	73.2 (7.2)	73.9 (6.6)	12.5 (2.8)	13.0 (3.5)	NA	NA	11.5
Hoppstädter et al. (2013), Serra et al. (2013)	10 (6/4)	14 (4/10)	67.8 (4.7)	68.0 (4.0)	12.90 (3.80)	11.30 (2.50)	28.88 (1.05)	27.85 (1.29)	11.5
Serra et al. (2013), Zhao et al. (2014)	28 (10/18)	15 (4/11)	63.4 (8.9)	70.9 (9.0)	13.1 (3.5)	11.3 (4.4)	28.4 (1.7)	25.4 (1.7)	11
Zhao et al. (2014), Hong et al. (2015)	18 (10/8)	20 (12/8)	66.8 (7.4)	65.1 (9.9)	12.0 (2.9)	11.8 (3.3)	29.3 (1.2)	25.2 (2.2)	11.5
Hong et al. (2015), Migo et al. (2015)	28 (19/9)	29 (19/10)	70.6 (6.5)	70.5 (5.2)	8.8 (6.16)	8.6 (4.36)	28.7 (1.36)	25.5 (2.81)	11.5
Migo et al. (2015)	11 (4/7)	10 (5/5)	70.3 (6.2)	71.4 (6.4)	15.64 (4.13)	16.00 (4.30)	NA	NA	10.5
Xie et al. (2015), Xie et al. (2015), Zhao et al. (2015)	26 (12/14)	30 (11/19)	64.8 (7.59)	67.14 (9.3)	14.3 (3.2)	14.3 (3.2)	28.2 (1.3)	27.1 (1.6)	11.5
Zhao et al. (2015), Sheelakumari et al. (2018)	34 (16/18)	34 (20/14)	66.9 (6.7)	68.0 (7.6)	11.5 (3.9)	10.8 (3.3)	29.2 (0.9)	25.5 (1.6)	11.5
Sheelakumari et al. (2018), Chen et al. (2020)	25	24	63.24 (6.94)	69.8 3(5.76)	12.80 (3.68)	11.29 (3.25)	NA	NA	11.5
Chen et al. (2020)	29 (17/12)	20 (7/13)	70.69 (5.4)	71.35 (5.9)	12.17 (3.2)	10.88 (2.9)	28.55 (1.4)	27.45 (2.1)	11.5

*aMCI, amnestic mild cognitive impairment; SD, standard deviation; MMSE, mini-mental state examination; NA, not available; F, female; and M, male*.

### Gray Matter Atrophy

**Figure 2:**
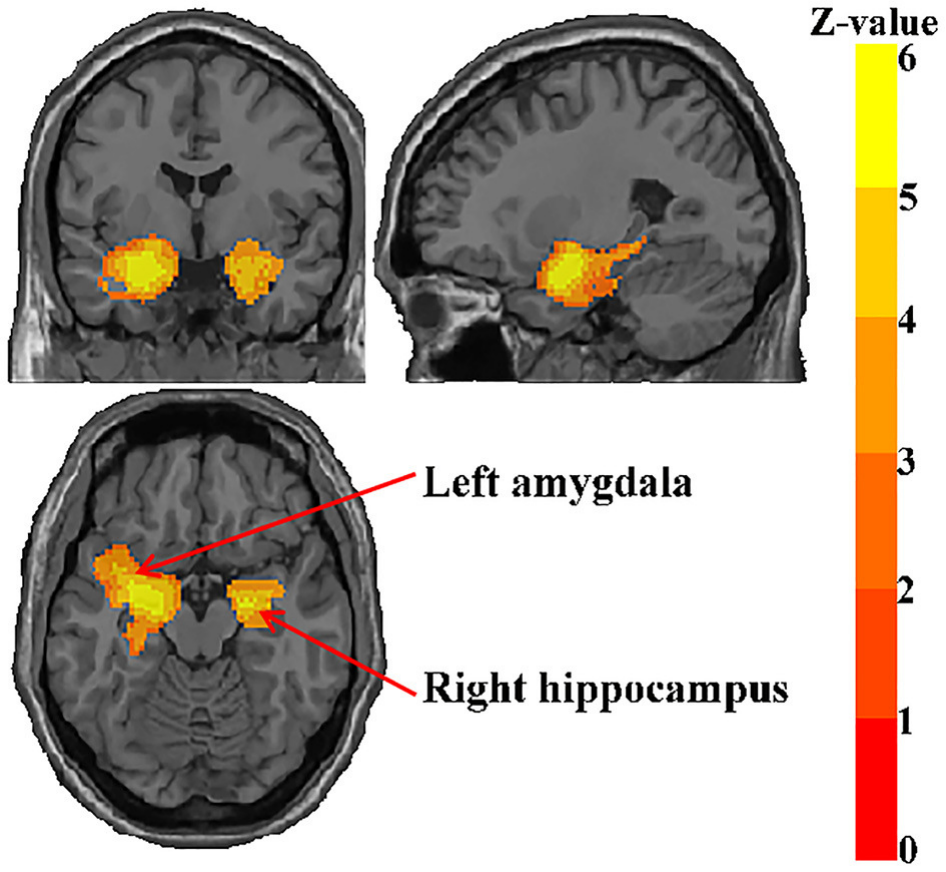
Regions showing reduced gray matter in aMCI patients.

In the pooled meta-analysis, aMCI patients showed significant gray matter atrophy in two brain regions relative to HC; one in the left amygdala (*p* = 0.000999987 < 0.001) extending to the left hippocampus, left temporal pole, superior temporal gyrus, left amygdala, cingulum, and left parahippocampal gyrus. The other in the right hippocampus (*p* = 0.000999987 < 0.001), extending to the right amygdala and right parahippocampal gyrus (Figure 2; Table 2).

**Table 2:** Regional differences in GM volume between patients with aMCI and healthy controls in the meta-analysis.

**Brain regions**	**MNI coordinate**	**SDM-Z value**	***P*-value**	**Number of voxels**	**Cluster breakdown** ** (number of voxels)**	**Jackknife sensitivity**
Left amygdala	−26, −2, −16	−6.635	<0.001	2,633	Left hippocampus, BA 20 (177); left temporal pole, superior temporal gyrus, BA 38 (165); left median network, cingulum (128); left parahippocampal gyrus, BA 28 (125); left inferior network, inferior longitudinal fasciculus (112); left inferior network, uncinate fasciculus (94); left insula, BA 48 (91);	31/31
Right hippocampus	20, −6, −14	−5.973	<0.001	836	Right amygdala, BA 34 (86); right parahippocampal gyrus, BA 28 (66)	30/31

*BA, Brodmann area*.

### Jackknife Sensitivity Analysis and Subgroup Analysis

Whole-brain jackknife sensitivity analysis showed that gray matter atrophy in the left amygdala and right hippocampus were highly replicable (Table 2). The results of the right amygdala and left thalamus remained significant in all but one combination (Trivedi et al., 2006).

The results remained largely unchanged when the meta-analysis was restricted to the datasets corrected for multiple comparisons (*n* = 14). However, when the meta-analysis was restricted to the datasets acquiring images with a 3.0 T MRI scanner (*n* = 16), datasets using statistical parametric mapping (SPM) software, versions 8 or 12 (*n* = 19), only the left amygdala was found (Table 3).

**Table 3:** Results of analyses of subgroups.

	**Decreased gray matter**	
	**Left amygdala**	**Right hippocampus**
Studies Hämäläinen et al., 2007; Barnes et al., 2009; Threlkeld et al., 2011; Venneri et al., 2011; Baglio et al., 2012; Han et al., 2012; Wang et al., 2012; Bastin et al., 2013; Hoppstädter et al., 2013; Serra et al., 2013; Zhao et al., 2014; Hong et al., 2015; Migo et al., 2015; Sheelakumari et al., 2018 with acquiring images with a 3.0T MRI scanner (*n* = 14)	Yes	No
Studies Barnes et al., 2009; Venneri et al., 2011; Wang et al., 2012; Bastin et al., 2013; Hoppstädter et al., 2013; Serra et al., 2013; Zhao et al., 2014, 2015; Migo et al., 2015; Sheelakumari et al., 2018; Chen et al., 2020 employing statistical parametric mapping (SPM) software, versions 8 or 12 (*n* =11)	Yes	No
Studies Bell-McGinty et al., 2005; Saykin et al., 2006; Trivedi et al., 2006; Bai et al., 2008; Barbeau et al., 2008; Guedj et al., 2009; Pa et al., 2009; Rami et al., 2009; Agosta et al., 2011; Derflinger et al., 2011; Threlkeld et al., 2011; Han et al., 2012; Wang et al., 2012; Xie et al., 2012; Bastin et al., 2013; Zhao et al., 2014, 2015 with correction for multiple comparisons (*n* = 16)	Yes	Yes

### Analyses of Heterogeneity and Publication Bias

The low *I*^2^ statistic (0.0%) (Supplementary Figures 1, 2) indicated low heterogeneity between-study variability in gray matter atrophy in the right hippocampus and left amygdala.

**Figure 3:**
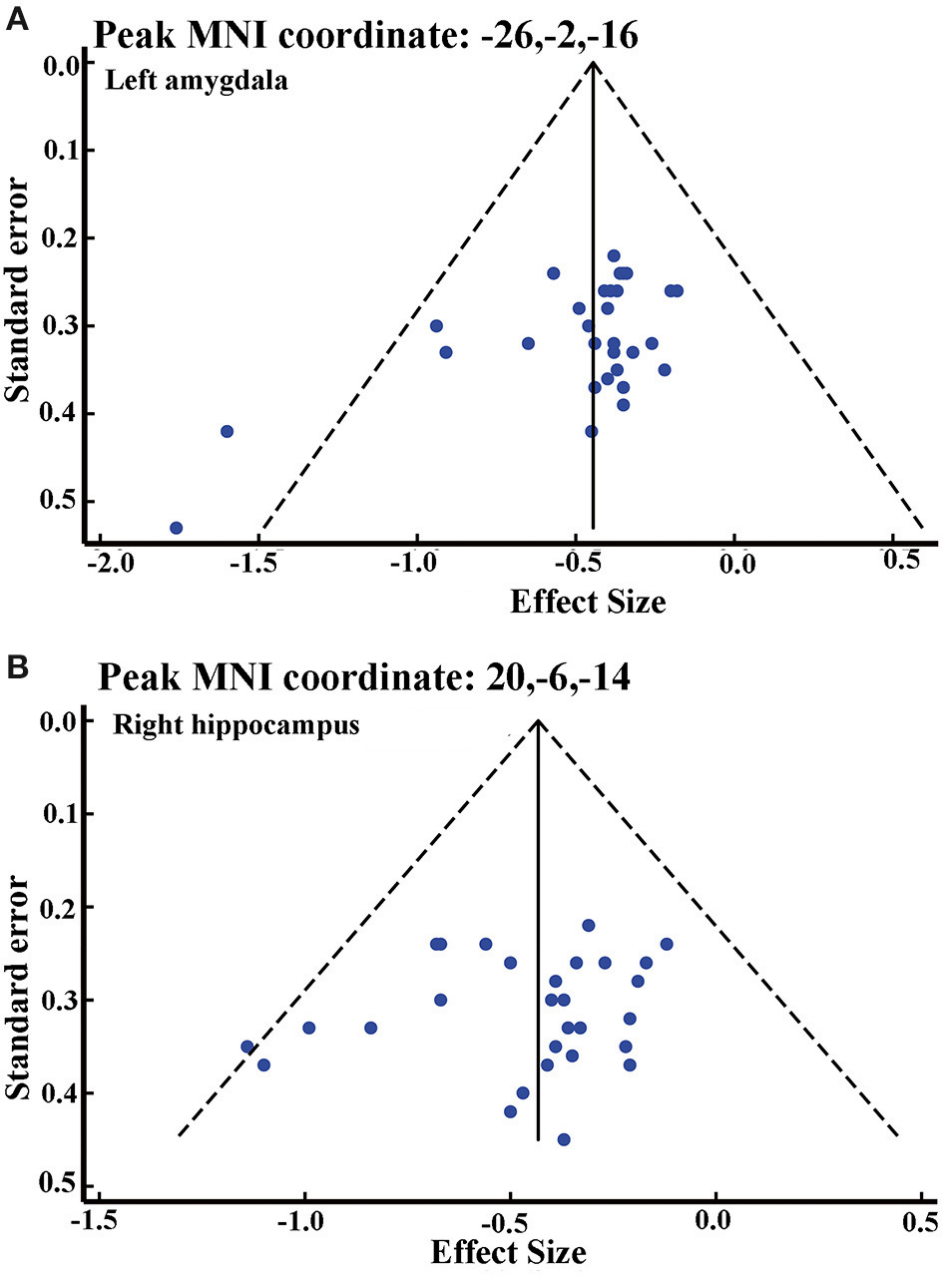
Funnel plot of effect size of left amygdala **(A)** and right hippocampus **(B)**.

Although the funnel plot showed no obvious asymmetry for the right hippocampus and the left amygdala (Figure 3), Egger tests revealed possible publication bias in the left amygdala (*p* < 0.05) (Supplementary Table 4).

### Meta-Regression Analysis

**Figure 4:**
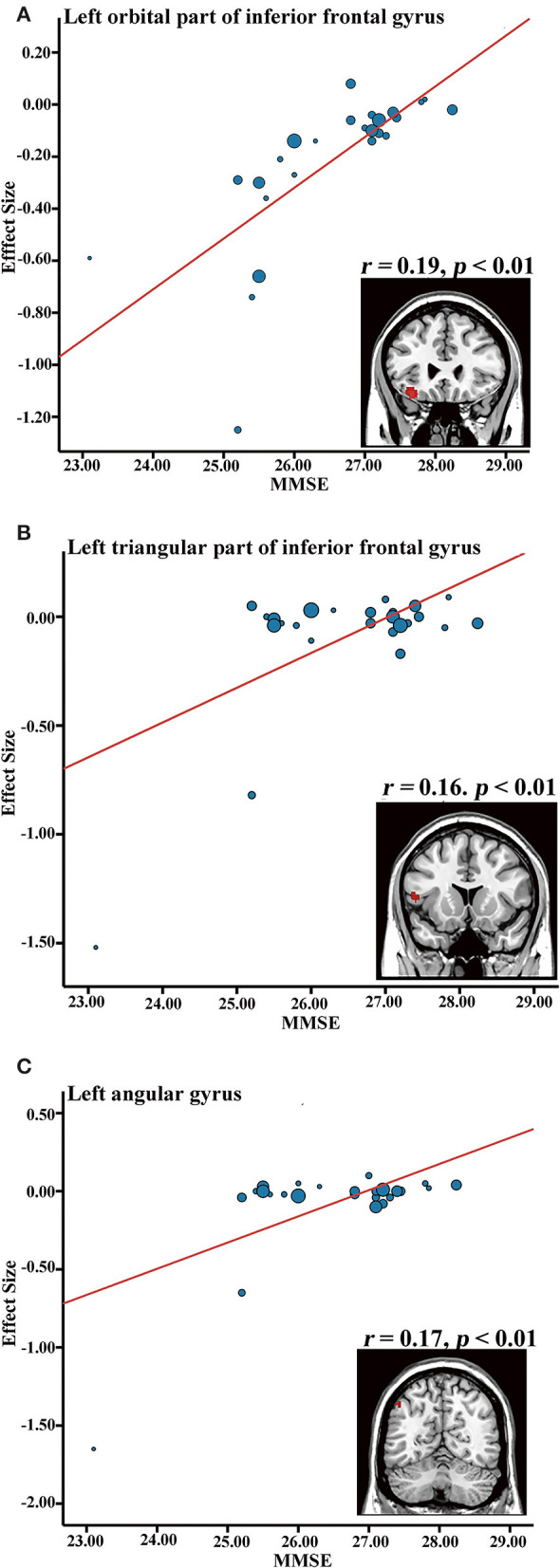
Meta-regression results showing an association between MMSE and gray matter volume in the aMCI group, in the left inferior frontal gyrus **(A)**, left inferior frontal gyrus **(B)**, and left angular gyrus **(C)**. MMSE, mini-mental state examination.

GMV atrophy in the left orbital part of the inferior frontal gyrus (IFG.L) (BA 47, MNI coordinate: *x* = −36, *y* = 26, *z* = −16, SDM-Z value = 2.469, *r* = 0.19, *p* = 0.006, and 112 voxels), the left triangular part of the inferior frontal gyrus (BA 48, MNI coordinate: *x* = −52, *y* = 16, *z* = 8; SDM-Z value = 1.965, *r* = 0.16, *p* = 0.024, 47 voxels), and the left angular gyrus (AG.L) (BA 39, MNI coordinate: *x* = −48, *y* = −62, *z* = 48, SDM-Z value = 2.037, *r* = 0.17, *p* = 0.020, 19 voxels) was found to be positively correlated with the MMSE scores in the aMCI patients after removing the covariates of age and education (Figure 4).

### Results From ADNI

A total of 144 patients with aMCI (82 male/62 female, mean age = 74.97, mean MMSE = 29.64) and 83 HC (38 male/45 female, mean age = 75.90, mean MMSE = 26.85) were included in the current study. No significant difference was reported for age and gender between both groups (*p* > 0.05). The aMCI group showed significantly higher MMSE compared with the HC group (*p* < 0.01).

VBM analysis revealed that there were two clusters with a statistical significant difference: the left hippocampus (MNI coordinate: *x* = −25.5, *y* = −9, *z* = −16.5, *t* = 4.450, 2,609 voxels) and right amygdala (peak MNI coordinate: *x* = 21, *y* = −4.5, *z* = −18, *t* = 4.453). These results showed high overlap with the meta-analysis results (Supplementary Figure 3).

### Co-activation Patterns and Functional Characterization

To further investigate the role of significance clusters (the left amygdala and right hippocampus) at the functional network level, MACM analysis was performed; at the same time, functional characterizations was performed to explore the detailed functions and behavioral profiles of the left amygdala and the right hippocampus.

**Figure 5:**
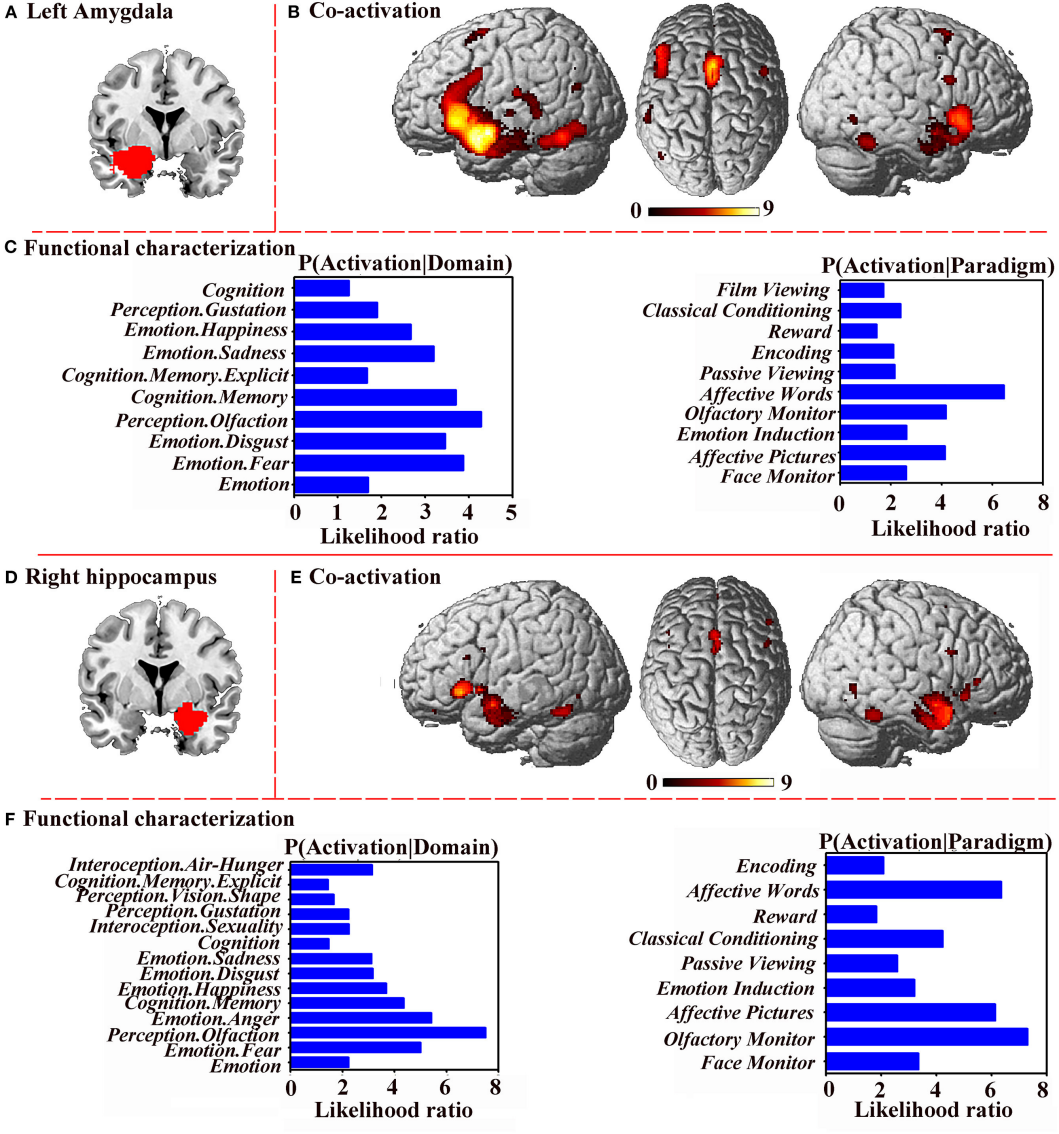
Functional connectivity and characterization. **(A)** Left amygdala. **(B)** Co-activation connectivity analysis for left amygdala. **(C)** Functional characterization of left amygdala. **(D)** Right hippocampus. **(E)** Co-activation connectivity analysis for right hippocampus. **(F)** Functional characterization of right hippocampus. Functional characterization of significance levels was thresholded at *p* < 0.05, cluster-level FWE-corrected, cluster-forming threshold at voxel-level *p* < 0.001. Co-activation connectivity of significance levels was thresholded at *p* < 0.05, corrected for multiple comparisons using FDR.

The bilateral cerebrum, limbic lobe, parahippocampal gyrus, amygdala, inferior frontal gyrus, sub-lobar, frontal lobe, inferior frontal gyrus, temporal lobe, extra-nuclear, superior frontal gyrus, and fusiform gyrus were co-activated with the left amygdala (Figures 5A,B). Functional characterization showed that the left amygdala was mainly associated with emotion, perception, and cognition, and the PCs showed similar results (Figure 5C).

The bilateral cerebrum, limbic lobe, parahippocampal gyrus, amygdala, temporal lobe, inferior frontal gyrus, sub-lobar, extra-nuclear, fusiform gyrus, and cingulate gyrus were co-activated with the right hippocampus (Figures 5D,E). Functional characterization demonstrated that the right hippocampus was associated with emotion, perception, and cognition, and the PCs also showed similar results (Figure 5F).

## Discussion

In the current study, a newly improved SDM-PSI method was used to perform a meta-analysis on VBM studies on GMV alterations in aMCI compared to HC. This quantitative meta-analysis comprised 31 whole-brain VBM studies with 681 aMCI patients and 837 HC. aMCI patients were found to exhibit significant gray matter atrophy in the left amygdala and the right hippocampus compared to HC. Jackknife sensitivity analysis and subgroup analysis revealed that the results were highly consistent and robust. Moreover, VBM analyses based on the ADNI dataset showed similar results with high overlap. Further meta-regression analysis demonstrated that GMV atrophy in the left inferior frontal gyrus and left angular gyrus is associated with the severity of cognitive impairment in aMCI. Functional characterization revealed that these regions were mainly associated with emotion, cognition, and memory.

Our findings of robust GMV atrophy in the left amygdala and right hippocampus were consistent with a previous meta-analysis based on ALE studies which reported evidence of volume reduction in the bilateral amygdala and hippocampus in aMCI (Albajes-Eizagirre et al., 2019b). Our results were also consistent with various longitudinal studies which showed that subjects with AD have higher rates of hippocampal and amygdala volumetric atrophy compared with HC (Jack et al., 1998; van de Pol et al., 2007; Wolz et al., 2010; Zhang et al., 2012; Miller et al., 2013). Moreover, the results were also supported by the previous meta-analysis between MCI and HC (Yang et al., 2012; Minkova et al., 2017; Gu and Zhang, 2019), however, there were some statistically significant clusters not reported in this study, which may be related to a heterogeneous subgroup of MCI patients (that is, aMCI and non-aMCI) (Hirata et al., 2005). These differences may be related to risk factors or physiology that is unique to each MCI subtype. Previous studies demonstrate that gray matter atrophy in MCI subtypes differs from HC (Whitwell et al., 2007; Bai et al., 2009a), aMCI showed more atrophy in the hippocampus, parahippocampus, and temporal lobes, whereas non-aMCI showed atrophy in the inferior and medial frontal gyrus, anterior cingulate gyrus, superior temporal gyrus, and insula (Whitwell et al., 2007). Most importantly, functional MRI studies report that aMCI patients show decreased medial temporal lobe activation (Chen et al., 2016) and decreased sub-regional functional connectivity (Remondes and Schuman, 2004; Bai et al., 2009b) compared with HC. It is important to note that the amygdala and hippocampus are two important medial temporal lobes (MTL) structures. MTL, involved in encoding and retrieval of episodic and spatial memory (Braak and Braak, 1991; Schwindt and Black, 2009; Ranganath and Ritchey, 2012), is considered to be initially targeted in AD-related pathology (Braak and Braak, 1991; Ranganath and Ritchey, 2012) and aMCI patients (Scheltens et al., 1992; Karas et al., 2004; Korf et al., 2004; Bai et al., 2009a). The results indicate that GMV atrophy in the left amygdala and right hippocampus might provide accurate potential biomarkers for aMCI.

This meta-analysis revealed GMV atrophy of the amygdala in aMCI, which has been reported in numerous other studies (Maren and Fanselow, 1995; Trivedi et al., 2006; Bastin et al., 2013; Migo et al., 2015). The important role of the amygdala in emotional processing and emotional memory has been emphasized by functional imaging experiments and lesion studies in animal models (Roozendaal et al., 2009; Pape and Pare, 2010; Mendez-Bertolo et al., 2016). Human studies further demonstrate the central roles of the amygdala in emotion processing (Adolphs et al., 1999; Sotres-Bayon et al., 2012), memory, and storage. Growing evidence suggests that aMCI patients have impaired recognition of facial emotional expression (McCade et al., 2011; Richard-Mornas et al., 2012; Varjassyova et al., 2013), which is mainly related to the amygdala. Results of our behavioral analysis for the amygdala also support this opinion. More importantly, impairments of emotional recognition and emotional facial expressions have been reported in aMCI patients (Lavenu et al., 1999), and are related to the transition of aMCI into AD (Bediou et al., 2009; Chen et al., 2015). Based on these findings, amygdala atrophy may help explain the clinical manifestations of aMCI.

Consistent with previous studies in aMCI patients (Maren and Fanselow, 1995; Apostolova et al., 2006; Shi et al., 2009; Bartsch and Wulff, 2015), this meta-analysis also showed GMV atrophy in the hippocampus, which is the most validated, easily accessible, and widely used biomarker for AD. The hippocampus, located in the MTL, plays a pivotal role in the learning, formation, and consolidation of memory (Knierim, 2015). Lesion studies on humans and animals demonstrate that the hippocampus performs a critical function in the brain's ability to store and retrieve memories (particularly episodic memories in humans) (Kontaxopoulou et al., 2018). Recent findings indicate that aMCI patients have difficulties with episodic memory, incidental memory, and long-term memory with greater hippocampal atrophy (Lee et al., 2014; Zhao et al., 2014; de Mendonca et al., 2018). Lee's study also showed that recognition memory can be used to identify aMCI patients at greater risk for progressing to dementia (Leung et al., 2013). These findings are consistent with our functional characteristics findings. An important study by Leung et al. found that hippocampal atrophy in MCI patients was estimated to accelerate by an average of 0.22%/year^2^ (Defrancesco et al., 2014). Therefore, our findings on hippocampal atrophy in patients with aMCI further emphasize the important role of the hippocampus in the pathobiology of aMCI.

MMSE, a general cognitive screening test commonly used to assess MCI in previous studies, has been correlated with GMV atrophy. In this study, lower MMSE was positively correlated with decreased GMV in the IFG.L, which was consistent with previous studies (Prince et al., 2005; Wang et al., 2012). The IFG.L has been implicated as an important part in the pathology of MCI, and is thought to be associated with attention and memory processes, including encoding and retrieval and long- and short-term memory (Wagner et al., 2001; Chambers et al., 2004; Prince et al., 2007). Similarly, lower MMSE was positively correlated with decreased GMV in the AG.L, which plays a major role in spatial attention and orienting (Dehaene et al., 2004), mathematical cognition (Thakral et al., 2017), and especially, episodic simulation and episodic memory (Bokde et al., 2010). Previous studies have shown that lower resting-state activity in the angular gyrus in aMCI may be related to poorer verbal working memory performance that involves short-term storage and retrieval of phonological representations (Jonides et al., 1998; Lin et al., 2020). These findings indicated that the IFG.L and AG.L may be used as potential markers to monitor aMCI progression and cognitive decline. Subgroup meta-analyses revealed that the main results were affected by the MR field-strength and SPM software to some extent, whereas GMV atrophy in the left amygdala and right hippocampus were independent of correction methods. These results provide insights to future VBM investigations, indicating the need to control for the potentially confounding factors of MR field-strength and SPM software.

The current study has several strengths. The most important one is the use of SDM-PSI, an updated CBMA, which has been presented and recommended in several previous studies (Yu et al., 2017; Albajes-Eizagirre et al., 2019b; Sheng et al., 2020a,b; Wang et al., 2020). This technique has made significant methodological improvements to overcome the drawbacks of alternative procedures and produce accurate results (Albajes-Eizagirre et al., 2019a). Besides, functional characterization and task-based co-activation using the BrainMap database was performed to explore the functional roles of the abnormal regions between aMCI and HC. Finally, the ADNI database was used to investigate the reliability of our findings.

Despite these strengths, this study has several potential limitations. First, the study shows that there may be publication bias in the gray matter atrophy of the amygdala. This may be related to the fact that we include only studies published in English that have been peer-reviewed. However, one study performed by Yu et al. also showed that publication bias did not have a major influence on the results in general (Salimi-Khorshidi et al., 2009). Our VBM results from the ADNI database showed similar patterns with those of the meta-analysis, which further supports the findings. Future comprehensively pooled big neuroimaging data from worldwide populations is still warranted. Second, voxel-based meta-analyses are based on summarized coordinates from published studies rather than raw data, which may result in less accurate results (Li and Zhang, 2015). However, obtaining and analyzing the raw images from these studies is logistically and technically difficult. Third, we did not perform subgroup analyses on the aMCI-single domain (aMCI-sd) and aMCI-multiple domain (aMCI-md), yet, distinct clinical features of aMCI subtypes may indicate different conversion rates to AD (Li and Zhang, 2015). Finally, since MMSE scores are a rather unspecific measure for aMCI (Nickl-Jockschat et al., 2012), the relationship between MMSE and brain structure should be used with caution. More specific memory tests are needed in future studies to better explore the relationship between gray matter atrophy and cognitive impairment in aMCI.

## Conclusions

The current meta-analysis supports that GMV atrophy in the left amygdala and right hippocampus is highly consistent in aMCI patients. Additionally, functional characterization demonstrates that the consistent regions of brain atrophy are functionally linked to “emotion,” “perception,” and “cognition.” This not only offers a better understanding of the underlying neuropathology but also provides accurate potential biomarkers for aMCI.

## Data Availability Statement

The original contributions presented in the study are included in the article/Supplementary Material, further inquiries can be directed to the corresponding authors.

## Author Contributions

JZ and JX designed the whole study, analyzed the data, and wrote the manuscript. XH and JL searched for and selected the studies. YL and FY participated in the interpretation of data. KL and YH participated in the revision of the article. HY and QH offered good suggestions. All authors read and approved the final manuscript.

## Conflict of Interest

The authors declare that the research was conducted in the absence of any commercial or financial relationships that could be construed as a potential conflict of interest.
