# Supplementary material for: Gray Matter Atrophy in Amnestic Mild Cognitive Impairment: A Voxel-Based Meta-Analysis
Source: Front Aging Neurosci. 2021 Mar 31;13:627919. doi: 10.3389/fnagi.2021.627919 (PMC8044397; doi:10.3389/fnagi.2021.627919)
Supplement: Supplementary file 1 [file Table_1.DOCX]

**Supplementary Materials**

**Consistent gray matter atrophy in amnestic mild cognitive impairment**Jinhuan Zhang^1,2^, Yongfeng Liu^3^, Xingxian Huang^3^, Fuxia Yang^3^,Jiaying Li^2^, Qingmao Hu^2^, Haibo Yu^1,3,*^, Jinping Xu^2,*^

^1^The fourth Clinical Medical College of Guangzhou University of Chinese Medicine, Shenzhen, 518033,China

^2^Institute of Biomedical and Health Engineering, Shenzhen Institutes of Advanced Technology, Chinese Academy of Sciences, Shenzhen 518055, China

^3^Shenzhen Traditional Chinese Medicine Hospital, Shenzhen, 518033,China

**Contents**

TableS1. Checklist of the PRISMA extension for network meta-analysis.........................................2

[TableS2. Imaging Methodology Quality Assessment Checklist (When criteria were partially met, 0.5 points were assigned) 6](#_Toc1287)

[Table S3.Technique details of VBM studies for GMV on aMCI in meta-analysis 7](#_Toc27632)

FigureS1.Forest plot of meta-analysis for effect size of left amygdala............................................11

[FigureS2. Forest plot of meta-analysis for effect size of right hippocampus 12](#_Toc2414)

[Table S4.Egger's test 12](#_Toc10104)

**TableS1. Checklist of the PRISMA extension for network meta-analysis**

| **Section/Topic** | **#** | **Checklist Item** | **Reported on Page #** |
| --- | --- | --- | --- |
| **TITLE** | | | |
| Title | 1 | Identify the report as a systematic review, meta-analysis, or both. | 1 |
| **ABSTRACT 2** | | | |
| Structured summary | 2 | Provide a structured summary including, as applicable: background; objectives; data sources; study eligibility criteria, participants, and interventions; study appraisal and synthesis methods; results; limitations; conclusions and implications of key findings; systematic review registration number. | 2 |
| **INTRODUCTION** | | | |
| Rationale | 3 | Describe the rationale for the review in the context of what is already known. | 3-4 |
| Objectives | 4 | Provide an explicit statement of questions being addressed with reference to participants, interventions, comparisons, outcomes, and study design (PICOS). | 3-4 |
| **METHODS** | | | |
| Protocol and registration | 5 | Indicate if a review protocol exists, if and where it can be accessed (e.g., Web address), and, if available, provide registration information including registration number. | 4 |
| Eligibility criteria | 6 | Specify study characteristics (e.g., PICOS, length of follow-up) and report characteristics (e.g., years considered, language, publication status) used as criteria for eligibility, giving rationale. | 5 |
| Information sources | 7 | Describe all information sources (e.g., databases with dates of coverage, contact with study authors to identify additional studies) in the search and date last searched. | 4 |
| Search | 8 | Present full electronic search strategy for at least one database, including any limits used, such that it could be repeated. | 4 |
| Study selection | 9 | State the process for selecting studies (i.e., screening, eligibility, included in systematic review, and, if applicable, included in the meta-analysis). | 4 |
| Data collection process | 10 | Describe method of data extraction from reports (e.g., piloted forms, independently, in duplicate) and any processes for obtaining and confirming data from investigators. | 5 |
| Data items | 11 | List and define all variables for which data were sought (e.g., PICOS, funding sources) and any assumptions and simplifications made. | 4 |
| Risk of bias in individual studies | 12 | Describe methods used for assessing risk of bias of individual studies (including specification of whether this was done at the study or outcome level), and how this information is to be used in any data synthesis. | 5 |
| Summary measures | 13 | State the principal summary measures (e.g., risk ratio, difference in means). | NA |
| Synthesis of results | 14 | Describe the methods of handling data and combining results of studies, if done, including measures of consistency (e.g., I^2^) for each meta-analysis. | 5-7 |

| **Section/Topic** | **#** | **Checklist Item** | **Reported on Page #** |
| --- | --- | --- | --- |
| Risk of bias across studies | 15 | Specify any assessment of risk of bias that may affect the cumulative evidence (e.g., publication bias, selective reporting within studies). | 7 |
| Additional analyses | 16 | Describe methods of additional analyses (e.g., sensitivity or subgroup analyses, meta-regression), if done, indicating which were pre-specified. | 6-7 |
| **RESULTS** | | | |
| Study selection | 17 | Give numbers of studies screened, assessed for eligibility, and included in the review, with reasons for exclusions at each stage, ideally with a flow diagram. | 9 |
| Study characteristics | 18 | For each study, present characteristics for which data were extracted (e.g., study size, PICOS, follow-up period) and provide the citations. | Table1,TalbeS4 |
| Risk of bias within studies | 19 | Present data on risk of bias of each study and, if available, any outcome level assessment (see item 12). | Table1 |
| Results of individual studies | 20 | For all outcomes considered (benefits or harms), present, for each study: (a) simple summary data for each intervention group (b) effect estimates and confidence intervals, ideally with a forest plot. | 9 |
| Synthesis of results | 21 | Present the main results of the review. If meta-analyses done, include for each, confidence intervals and measures of consistency. | 9-12 |
| Risk of bias across studies | 22 | Present results of any assessment of risk of bias across studies (see Item 15). | NA |
| Additional analysis | 23 | Give results of additional analyses, if done (e.g., sensitivity or subgroup analyses, meta-regression [see Item 16]). | 13 |
| **DISCUSSION** | | | |
| Summary of evidence | 24 | Summarize the main findings including the strength of evidence for each main outcome; consider their relevance to key groups (e.g., healthcare providers, users, and policy makers). | 15-17 |
| Limitations | 25 | Discuss limitations at study and outcome level (e.g., risk of bias), and at review-level (e.g., incomplete retrieval of identified research, reporting bias). | 18 |
| Conclusions | 26 | Provide a general interpretation of the results in the context of other evidence, and implications for future research. | 18 |
| **FUNDING** | | | |
| Funding | 27 | Describe sources of funding for the systematic review and other support (e.g., supply of data); role of funders for the systematic review. | 19 |

*From:* Moher D, Liberati A, Tetzlaff J, Altman DG, The PRISMA Group (2009). Preferred Reporting Items for Systematic Reviews and Meta-Analyses: The PRISMA Statement. PLoS Med 6(6): e1000097. doi:10.1371/journal.pmed1000097

For more information, visit: **www.prisma-statement.org**

# TableS2. Imaging Methodology Quality Assessment Checklist (When criteria were partially met, 0.5 points were assigned)

|  |
| --- |
| **Category 1: Subjects Score (0/0.5/1)** |
| 1 Patients were evaluated prospectively, specific diagnostic criteria were applied, and demographic data was reported |
| 2 Healthy comparison subjects were evaluated prospectively, psychiatric and medical illnesses were excluded |
| 3 Important variables (e.g. age, gender, illness duration, onset time, medication status, comorbidity, severity of illness) were checked, either by stratification or statistically |
| 4 Sample size per group > 10 |
| **Category 2: Methods for image acquisition and analysis** |
| 5 Magnet strength at least 1.5T |
| 6 MRI slice-thickness≤3 mm |
| 7 Whole brain analysis was automated with no a-priori regional selection |
| 8 Coordinates reported in a standard space |
| 9 The imaging technique used was clearly described so that it could be reproduced |
| 10 Measurements were clearly described so that they could be reproduced |
| **Category 3: Results and conclusions** |
| 11 Statistical parameters for significant, and important non-significant, differences were provided |
| 12 Conclusions were consistent with the results obtained and the limitations were discussed |
| **TOTAL /12** |

# Table S3.Technique details of VBM studies for GMV on aMCI in meta-analysis

| **Study** | **MCI/HC** | **Scanner (Tesla)** | **Thickness (mm)** | **Algorithm** | **Stereotaxic space** | **Soft-ware** | **Modulation** | **Smoothing kernel (mm)** | **Covariates included** | **Significance level** |
| --- | --- | --- | --- | --- | --- | --- | --- | --- | --- | --- |
| Chetelat 2002^49^ | 22/22 | 1.5 T | NA | Unified | Talairach (Brett) | SPM99 | modulated | 12 | Age | *p* < 0.01 FDR |
| Bell-McGinty2005^50^ | 9/47 | 1.5 T | 1.5 | Unified | MNI | SPM99 | modulated | 8 | Age | *P* < 0.001 uncorrected |
| Hirata2005^51^ | 41/30 | 1.0-T | 1.23 | NA | Talairach | SPM2 | NA | 12 | NA | *p* = 0.01 with correction for multiple non-independent comparison |
| Saykin 2006^52^ | 40/40 | 1.5 T | NA | Optimized | MNI | SPM2 | unmodulated | 12 | Age, TIV | *p* < 0.001 uncorrected |
| Shiino2006^53^ | 20/88 | 1.5 T | 1.4 | Optimized | Talairach | SPM99 | unmodulated | 12 | NA | *p* < 0.05 FWE |
| Trivedi2006^54^ | 15/15 | 3.0 T | 1.2 | Optimized | MNI | SPM2 | modulated | 12 | TIV | *p* < 0.005 uncorrected |
| Hamalainen 2007^55^ | 14/21 | 1.5 T | 1 | Optimized | MNI | SPM2 | modulated | 12 | Age, gender, TIV | *p* < 0.05 corrected |
| Bai2008^56^ | 20/20 | 1.5 T | 2 | segmentation | Talairach | SPM5 | modulated | 4 | NA | *p* < 0.05 corrected for multiple comparisons |
| Barbeau2008^57^ | 28/28 | 1.5 T | 1.25 | Traditional | Talairach | SPM2 | modulated | 6 | Age | *p* < 0.05 FDR |
| Guedj2009^58^ | 29/28 | 1.5 T | 1.5 | Traditional | Talairach | SPM2 | modulated | 6 | Age | *p* < 0.05 FDR |
| Pa2009^59^ | 26/36 | 1.5 T | 1.5 | Unified | MNI | SPM5 | modulated | 12 | Age, gender, TIV | *p* < 0.05 FWE |
| Rami2009^60^ | 14/27 | 1.5T | 1.5 | NA | MNI | SPM2 |  | 10 | Age and sex | *p* < 0.001 uncorrected |
| Bonekamp2010^61^ | 10/20 | 1.5 T | 1.5 | Optimized | Talairach (Brett) | SPM2 | modulated | 8 | Gender | *p* < 0.05 FWE |
| Agosta2011^62^ | 15/15 | 1.5 T | 1 | Optimized | MNI | SPM5 | modulated | 12 | Age, gender, TIV, CDR | *p* < 0.005 FWE |
| Deflinger2011^63^ | 24/30 | 3.0 T | 1 | DARTEL | MNI | SPM8 | modulated | 8 | Age, gender, education | *p* < 0.05 corrected |
| Threlkeld2011^8^ | 18/24 | 3T | 1 | NA | MNI | SPM5 | modulated, | NA | Age, gender,  education, ICV | *p* < 0.05 FWE |
| Venneri2011^64^ | 25/25 | 3.0 T | 1 | Optimized | Talairach (Brett) | SPM5 | modulated | 6 | Age, gender education | *p* < 0.05 FWE |
| Baglio2012^7^ | 16/15 | 3-T | 1 | NA | MNI | SPM5 | modulated | 12 | NA | *p* < 0.005 uncorrected |
| Han2012^65^ | 17/18 | 3.0 T | 1 | DARTEL | Talairach | SPM8 | modulated | 8 | Age, gender, education | *p* < 0.05 FDR |
| Wang2012^66^ | 40/30 | 1.5-T | 1.5 | DARTEL | MNI | SPM5 | modulated | 8 | TIV, age, gender, and years of formal education | *p* < 0.05 FWE |
| Xie2012^67^ | 17/25 | 3.0 T | 1 | Optimized | MNI | SPM8 | modulation | 8 | Age, education, gender, and ICV | *p* < 0.01 FDR |
| Bastin2013^68^ | 35/24 | 3T | NA | NA | MNI | SPM8 | NA | NA | gender and total intracranial volume | *p* < 0.001 uncorrected |
| Hoppstädter2013^69^ | 14/10 | 3.0 T | 1 | DARTEL | MNI | SPM8 | modulated | 6 | TIV, age, gender | *p* < 0.005 uncorrected |
| Serra2013^70^ | 15/28 | 3.0 T | 1 | Unified | MNI | SPM8 | modulated | 12 | Age, gender, TIV, education | *p* < 0.05 FWE |
| Zhao2014^71^ | 20/18 | 3.0 T | 4 | NA | MNI | SPM5 | modulated | 4 | NA | *p* < 0.01 corr |
| Hong2015^72^ | 29/28 | 1.5 T | 1.2 | Optimized | MNI | SPM5 | modulated | 12 | Age, gender, education, TIV | *p* < 0.005uncorrected |
| Migo2015^6^ | 10/11 | 3.0 T | 3 | DARTEL | MNI | SPM8 | NA | 8 | Age and ICV | *p*  < 0.001 uncorrected |
| Xie2015^73^ | 30/26 | 1.5 T | 1 | Optimized | MNI | SPM8 | modulation | 8 | age, education, gender and group | *p* < 0.001FWE |
| Zhao2015^74^ | 34/34 | 3.0 T | 1 | Optimized | MNI | SPM8 | modulated | 8 | NA | *p* < 0.01 uncorrected |
| Sheelakumari2018^75^ | 24/25 | 1.5T | 1 | DARTEL | MNI | SPM8 | modulated | 8 | age,sex, and TIV | *p* < 0.001 corrected |
| Chen2020^76^ | 20/29 | 3.0 T | 1 | NA | MNI | SPM8 | modulated | 8 | age, gender, years  of education, and APOE | P<0.005 uncorrected |

Abbreviations: DARTEL, diffeomorphic anatomical registration through an exponentiated lie algebra; DPARSF, Data Processing Assistant for Resting-State; NA, Not Available; FWE, family-wise error; FDR, false discovery rate; ICV, intracranial volume; MNI, Montreal Neurological Institute; MR, magnetic resonance; SPM, Statistical Parametric Mapping; TIV, total intracranial volume;VBM, voxel-based morphometry.


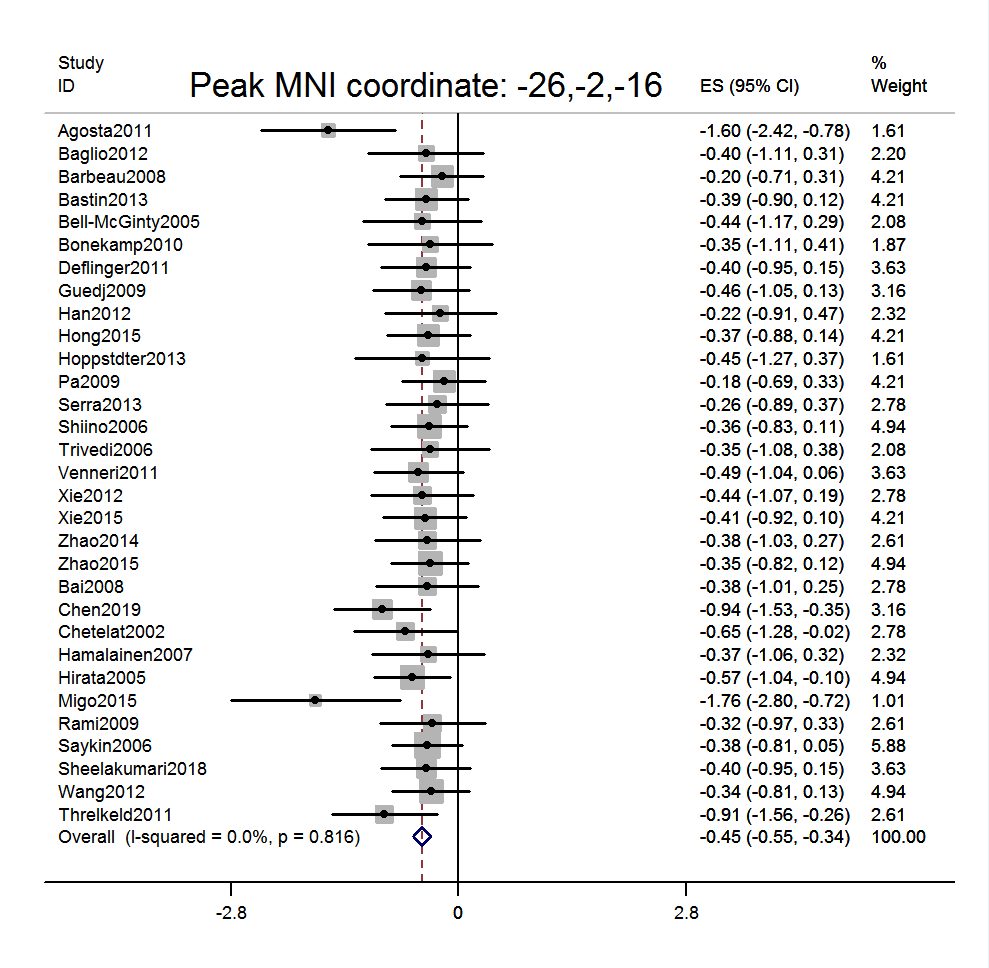
FigureS1. Forest plot of meta-analysis for effect size of left amygdala


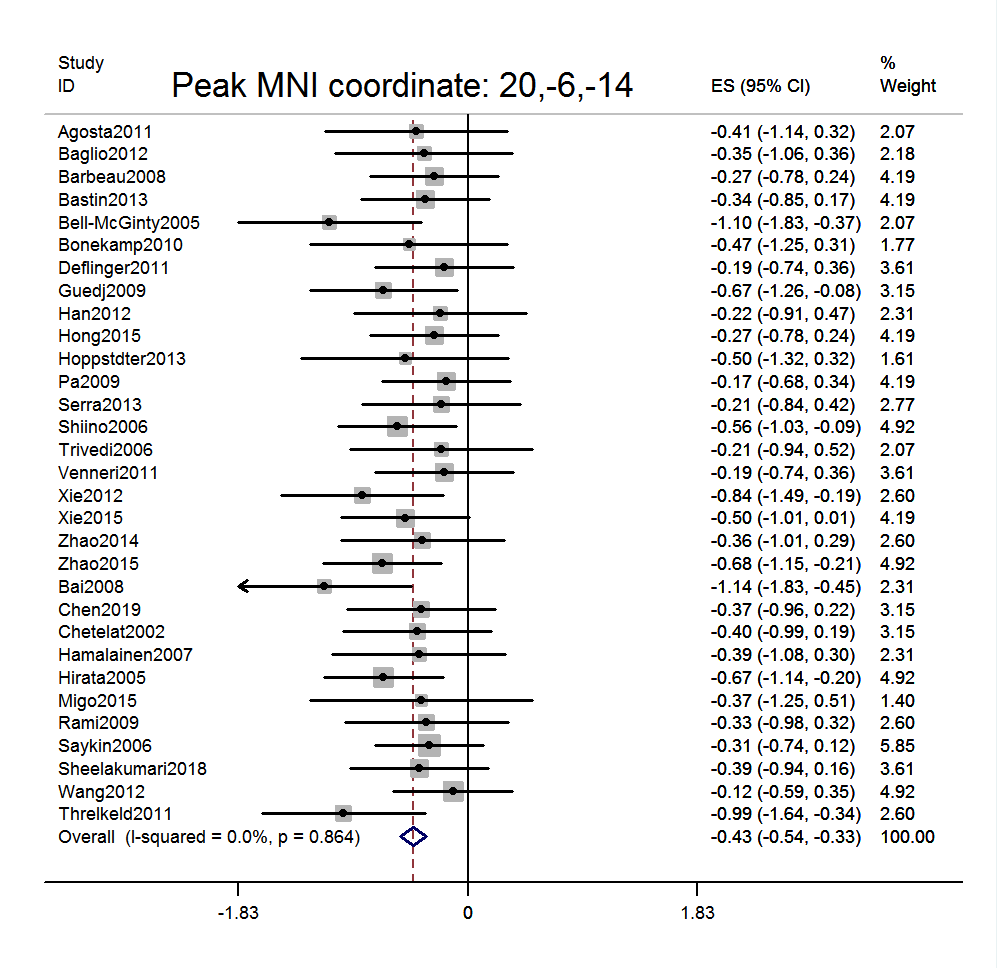


# FigureS2. Forest plot of meta-analysis for effect size of right hippocampus

# Table S4.Egger's test

Table S4a.Egger's test for left amygdala

| Std_Eff | Coef. | Std. Err. | t | P>\|t\| | [95% Conf. Interval] | |
| --- | --- | --- | --- | --- | --- | --- |
| slope | 0.1955287 | 0.224041 | 0.87 | 0.39 | -0.2626866 | 0.653744 |
| bias | -2.195979 | 0.7541907 | -2.91 | 0.007 | -3.738472 | -0.6534858 |

Table S4b.Egger's test for right hippocampus

| Std_Eff | Coef. | Std. Err. | t | P>\|t\| | [95% Conf. Interval] | |
| --- | --- | --- | --- | --- | --- | --- |
| slope | -0.1506435 | 0.2536628 | -0.59 | 0.557 | -0.6694422 | 0.3681551 |
| bias | -0.9667517 | 0.8561517 | -1.13 | 0.268 | -2.717779 | 0.7842753 |





1. **Results of meta**
2. **Results of ADNI**

**Figure S3. The overlap between the results of ADNI and meta analysis.**
